# The human milk endocannabinoidome is preserved by high hydrostatic pressure processing but altered by holder pasteurization

**DOI:** 10.3389/fped.2025.1558524

**Published:** 2025-03-11

**Authors:** Lucie Marousez, Elizabeth Dumais, Léa Chantal Tran, Laure Dubernat, Marie De Lamballerie, Frédéric Gottrand, Delphine Ley, Nicolas Flamand, Vincenzo Di Marzo, Jean Lesage

**Affiliations:** ^1^ Univ. Lille, Inserm, CHU Lille, U1286—INFINITE—Institute for Translational Research in Inflammation, Lille, France; ^2^ Canada Research Excellence Chair in the Microbiome-Endocannabinoidome mediators Axis in Metabolic Health (CERC-MEND), Université Laval, Québec City, QC, Canada; ^3^ Centre de Recherche de L'Institut Universitaire de Cardiologie et de Pneumologie de Québec (IUCPQ), Université Laval, Québec City, QC, Canada; ^4^ Department of Paediatrics, Division of Gastroenterology, Hepatology and Nutrition, Jeanne de Flandre Children’s Hospital, CHU Lille, Lille, France; ^5^ GEPEA, UMR CNRS 6144, ONIRIS CS82225, Nantes, France; ^6^ Département de Médecine, Faculté de Médecine, Université Laval, Québec City, QC, Canada; ^7^ Joint International Research Unit for Chemical and Biomolecular Studies on the Microbiome and its Impact on Metabolic Health and Nutrition, Université Laval, Québec City, QC, Canada; ^8^ Institute of Biomolecular Chemistry, Consiglio Nazuionale Delle Ricerche, Pozzuoli, Italy; ^9^ Centre Nutrition, Santé et Société (NUTRISS), Institut sur la Nutrition et les Aliments Fonctionnels (INAF), Université Laval, Québec City, QC, Canada

**Keywords:** human milk, endocannabinoidome, high hydrostatic pressure, holder pasteurization, preterm infant

## Abstract

**Introduction:**

The microbiological safety of donor milk (DM) is commonly ensured by holder pasteurization (HoP, 62.5°C for 30 min) in human milk banks despite its detrimental effects on several bioactive factors. We compared the concentration of twelve endocannabinoid (eCB)-like mediators in raw DM and in DM after holder pasteurization or high hydrostatic pressure processing (HHP, 350 MPa at 38°C), a non-thermal substitute for DM pasteurization.

**Methods:**

We measured five N-acyl-ethanolamines (NAEs) and seven 2-mono-acyl-glycerols (2-MAGs) in raw-DM, HHP-DM and HoP-DM using high-performance liquid chromatography coupled to tandem mass spectrometry (LC-MS/MS).

**Results:**

HoP-DM and HHP-DM demonstrated comparable concentrations compared with raw DM of 2-MAGs as well as for N-docosapentaenoyl-ethanolamine (DHEA, an NAE). However, four other NAEs, including N-arachidonoyl-ethanolamine (AEA), N-palmitoyl-(PEA), N-oleoyl -ethanolamine (OEA) and N-linoleoyl-ethanolamine (LEA) were significantly increased by HoP.

**Conclusion:**

Our study suggests that HHP-DM may more suitable than HoP-DM to improve the development of preterm infants through the preservation of milk eCB mediators at level close to their initial levels in raw DM.

## 1 Introduction

Human milk is a nutritional fluid rich in lipids that provides a large part of energy requirements for neonates up to 6 months of age (1). Arachidonic acid is the most predominant long-chain polyunsaturated fatty acid in human milk and is essential for infant development (2). In addition to its direct biological effects, this fatty acid also serves as a precursor of others classes of lipids such as eicosanoids and endocannabinoids (eCBs) (3). The eCB system includes the two ligands, i.e., N-arachidonoyl-ethanolamine (anandamide, AEA) and 2-arachidonoyl-glycerol (2-AG), as well as their main molecular targets, the cannabinoid receptors (CB1 and CB2). However, several others congeners of AEA and 2-AG, such as the N-acyl-ethanolamines (NAEs) and 2-mono-acylglycerols (2-MAGs), which activate various non-cannabinoid receptors, have also been identified. The signalling system including eCBs, eCB-like molecules and their metabolic enzymes and molecular targets is now referred to as the endocannabinoidome (eCBome) (4). Although eCBome mediators have been identified in human milk by several groups (5–7), there is still limited information published defining their functions in infants. Recently, the association between the profile of some eCBome mediators in human milk and infant growth in the context of gestational diabetes has been reported suggesting a role of this system in the control of body growth postnatally (7). In adult rodents, eCBs have been shown to play a key role in appetite and food intake by activating CB1 in the central nervous system (8). In mouse pups, 2-AG, a potent CB1 agonist, has been shown to be implicated in the establishment of the suckling response by activating the oral-motor musculature behaviour needed for milk suckling (9, 10). The important role of CB1 activation in the control of body growth in newborns was further demonstrated by administration of a CB1 antagonist to mouse pups, which resulted in drastic body growth restriction and even death of pups eight days after birth (9). Thus, based on these findings, breast milk eCBome mediators may be implicated in infant suckling and postnatal development but also, as suggested in experimental studies related to the eCB system, in other physiological functions, including brain regulation of appetite and energy metabolism, pain-perception (11) and neuronal development (12).

Preterm infants have several immature organs such lung, intestine as well as central and peripheral nervous systems that expose them at risk of developing short- and long-lasting diseases (13). In some circumstances, mothers of preterm infants are not able to provide breast milk in sufficient quantity due to a foreshortened period of preparatory lactogenesis. Then, when the mother's own milk is not available, donor milk (DM) is the recommended alternative (14). Human milk banks (HMBs) provide pasteurized DM as alternative for feeding these fragile infants (14). Most HMBs pasteurize DM using the standard method of Holder pasteurization (HoP) performed by heating milk to 62.5°C for 30 min (15). However, in the last decade, numerous studies have reported that HoP reduces numerous important heat-sensitive bioactive compounds of DM such as immunoglobulins, lactoferrin, some vitamins, lysozyme, the bile salt-dependent lipase and several hormones (15–17). To prevent these alterations, new methods have been tested as an alternative to HoP such as the high hydrostatic pressure (HHP) processing. We and others have demonstrated that this non-thermal method is able to pasteurize DM and to prevent the degradation of almost all the important bioactive factors described previously to be reduced by HoP (17–21).

In human milk, lipids are packaged as milk fat globules, with unique fatty acid (FA) and triacylglycerol (TAG) profiles of a lipid core covered by a membrane mainly composed of polar lipids (22). Both HoP and HHP processing have been reported to alter some lipids in DM. For example, Ten-Doménech et al. (23) found that 289 lipids were significantly altered upon HoP and Tran et al. (21) observed that 113 lipids of human milk were modulated after a HHP processing of moderate intensity. As mammalian milk eCBome mediators are composed of particular bioactive lipids (24, 25), these compounds may also be affected by the pasteurization process of human milk. Thus, this study aims to evaluate the effect of DM pasteurization using HoP or HHP processing on the human milk concentrations of 12 eCBome mediators.

## 2 Materials and methods

### 2.1 Donor milk collection

The study protocol was approved by the ethics committee of the Groupe Francophone d'Hépatologie, Gastroentérologie et Nutrition Pédiatrique (2023-51). DM was collected from 11 volunteers in the regional HMB (Lactarium Régional de Lille, Jeanne de Flandre Children's Hospital, CHU Lille). All donors were fully informed about the research study and provided their written consent to participate.

### 2.2 Donor milk samples, HoP and HHP treatment

After thawing of milk samples, 7 different batches of DM were created under sterile conditions by mixing various volumes (from 10 to 30 ml) of all BM samples to homogenize DM composition among batches. Three aliquots of milk samples were prepared from each batch: one fraction was stored at −80°C without any other treatment [raw milk (RM) sample]; one fraction was subjected to HoP according to the standard pasteurization protocol in our regional HMB (62.5°C for 30 min); the last fraction was subjected to HHP processing as previously described (20, 21). Briefly, the HHP parameters were as follows: 350 MPa pressure, 38°C temperature, 1 MPa.s−1 VA (application rate), 4 cycles of 5 min each, and a latency time with normal pressure between each cycle of 5 min. After HoP and HHP processing, samples were stored at −80°C until analysis.

### 2.3 Analysis of eCBome mediators in donor milk

High-performance liquid chromatography coupled to tandem mass spectrometry (LC-MS/MS) was used to measure levels of NAEs and 2-MAGs in 50 ul in human milk sample (26). Briefly, For the analysis of MAGs and NAEs, milk samples were thawed and centrifuged (1,000 g; 10 min) to remove cellular debris, then supernatants were diluted with water to a final MeOH concentration of 10%, and maintained at pH 3 by the addition of acetic acid. Samples were loaded on solid phase extraction cartridges (Strata-X Polymeric Reversed Phase, 60 mg/1 ml, Phenomenex). Cartridges were washed with 2 ml of acidified water and lipids were eluted with 1 ml of MeOH. The eluates were evaporated to dryness under a stream of nitrogen. Lipids then were extracted from the denatured samples by adding 1 ml of chloroform, vortexing for 1 min, and centrifuging at 4,000 g for 5 min without brakes. This was repeated three times. The organic phases were collected, pooled, and evaporated to dryness under a stream of nitrogen. For the quantification of MAGs, samples were extracted as documented before (26). Samples were reconstituted in 25 µl of HPLC solvent A (H_2_O with 0.05% acetic acid and 1 mM NH4^+^) and 25 µl of solvent B (MeCN/H_2_O, 95/5, v/v, with 0.05% acetic acid and 1 mM NH4^+^). A 25 µl of aliquot was injected onto an RP-HPLC column (Kinetex C8, 150 × 2.1 mm, 2.6 µm, Phenomenex). Quantification was performed on a Shimadzu 8,050 triple quadrupole mass spectrometer. Quantification was achieved by generating calibration curves using pure standards and analyzed on the LC-MS/MS system three times.

The methods quantified five NAE mediators including: N-palmitoyl–ethanolamine (PEA), N-oleoyl–ethanolamine (OEA), N-linoleoyl–ethanolamine (LEA), N-arachidonoyl-ethanolamine (AEA), N-docosahexaenoyl-ethanolamine (DHEA), as well as seven 2-MAGs mediators including 2-palmitoyl-glycerol (2-PG), 2-oleoyl-glycerol (2-OG), 2-linoleoyl-glycerol (2-LG), 2-arachidonoyl-glycerol (2-AG), 2-eicosapenaenoyl-glycerol (2-EPG), 2-docosapentaenoyl-glycerol (2-DPG) and 2-docosahexaenoyl-glycerol (2DHG). The data are presented as 2-MAGs but represent the combination of the 2- and 1(3)-isomers because 1(3)-isomers are most likely generated from the former via acyl migration.

### 2.4 Statistical analysis

Data are presented as mean ± SEM. Statistical analysis were performed with GraphPad Prism 7.0. software (San Diego, USA). Grubb's test was used to detect any outliers. Normality of variables was evaluated by a d'Agostino-Pearson test. Statistical differences were then tested by Kruskal–Wallis (Dunn's post-test) according to sample normality assessment results. A *P* value <0.05 was considered significant.

## 3 Results

### 3.1 Effects of HoP and HHP treatment of DM on NAEs

**Figure 1:**
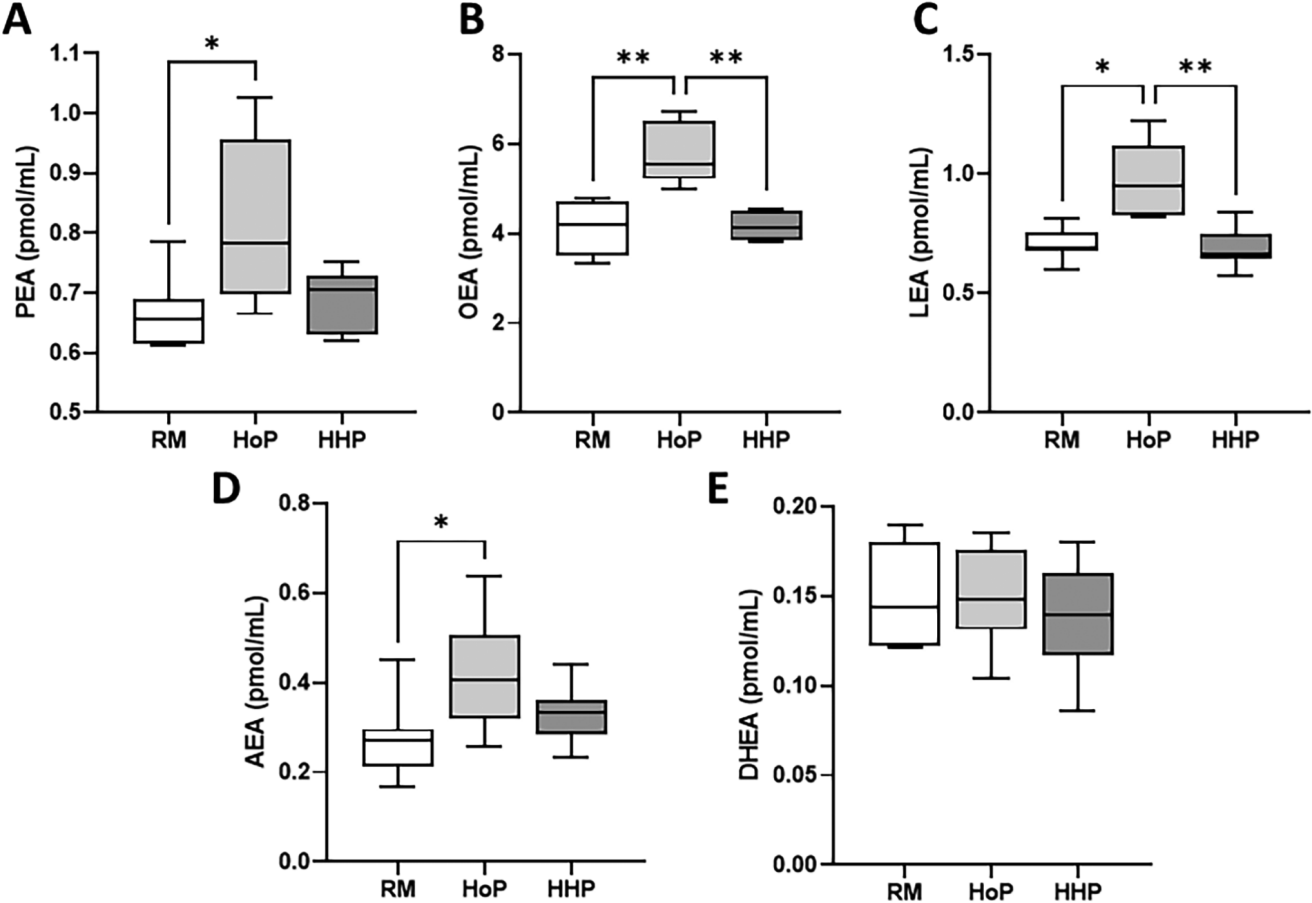
Concentrations of five NAEs in raw donor milk (RM) and after Holder Pasteurization (HoP) or High Hydrostatic Pressure (HHP) processing. **(A)** PEA, **(B)** OEA, **(C)** LEA, **(D)** AEA and **(E)** DHEA levels. Boxplots include the median, lower/higher quartiles and 1.5 x inter-quartile range whiskers. Statistically significant comparisons between HoP, HHP and RM conditions were made using Kruskal-Wallis with Dunn's post-test: *P < 0.05*, *P < 0.01.

Five NAE mediators were measured (Figure 1) namely PEA, OEA, LEA, AEA and DHEA. HoP treatment of DM significantly increased the concentration of four NAEs including PEA (+23%, Figure 1A), OEA (+42%, Figure 1B), LEA (+37%, Figure 1C) and AEA (+55%, Figure 1D) compared to their levels in raw DM. In contrast, HHP processing did not significantly affect the concentration of these compounds compared to raw DM (Figures 1A–D). DHEA concentration in DM was not affected by either pasteurization method (Figure 1E).

### 3.2 Effects of HoP and HHP treatments of DM on 2-MAGs

**Figure 2:**
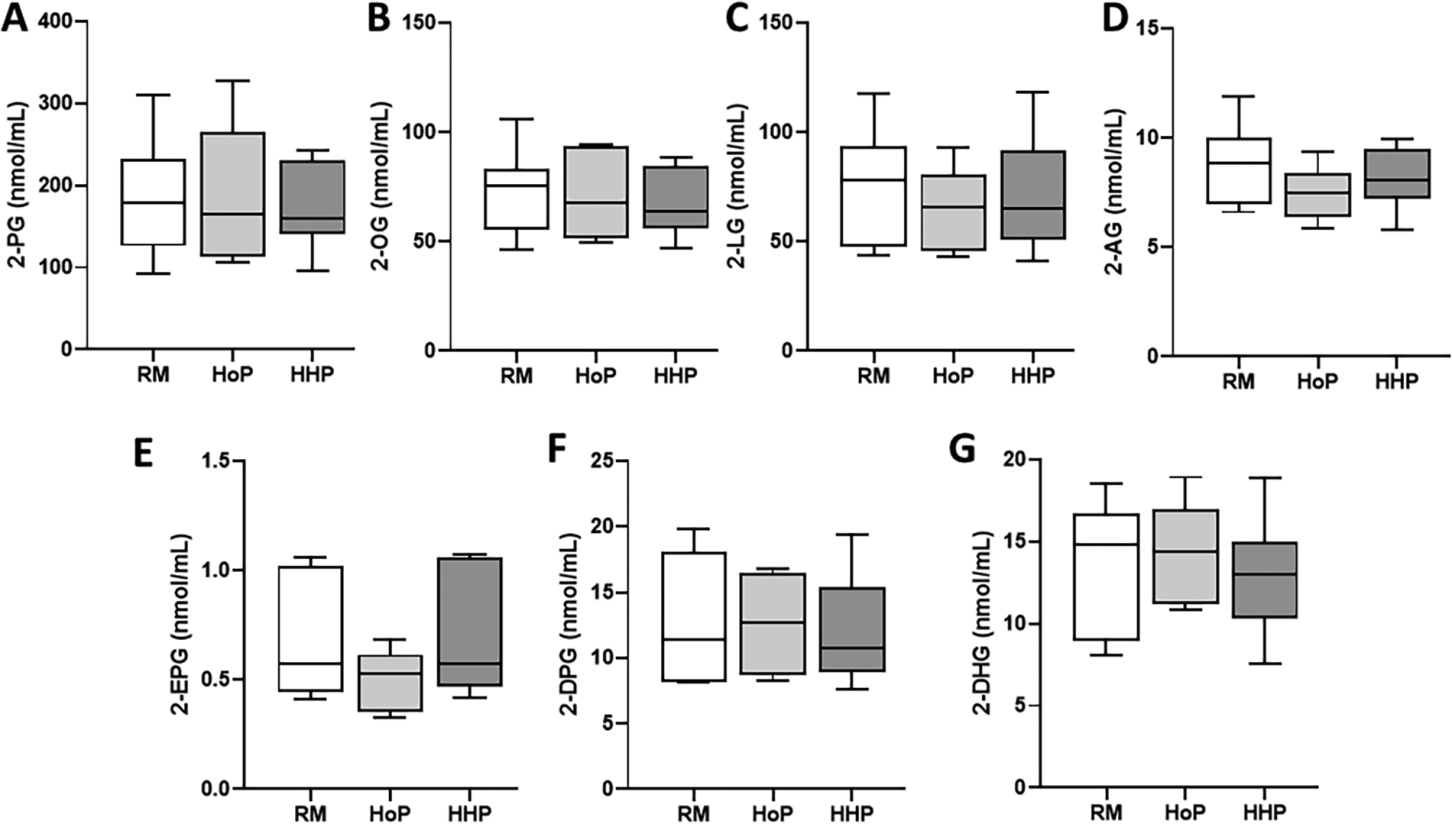
Concentrations of seven 2-MAGs in raw donor milk (RM) and after Holder Pasteurization (HoP) or High Hydrostatic Pressure (HHP) processing. **(A)** 2-PG, **(B)** 2-OG, **(C)** 2-LG, **(D)** 2-AG, **(E)** 2-EPG, **(F)** 2-DPG and **(G)** 2-DHG levels. Boxplots include the median, lower/higher quartiles and 1.5 x inter-quartile range whiskers. Statistically significant comparisons between HoP, HHP and RM conditions were made using Kruskal-Wallis with Dunn's post-test. No statical differences were found between experimental conditions.

Seven 2-MAGs were measured (Figure 2), including: 2-palmitoyl-glycerol (2-PG, Figure 2A), 2-oleoyl-glycerol (2-OG, Figure 2B), 2-linoleoyl-glycerol (2-LG, Figure 2C), 2-arachidonoyl-glycerol (2-AG, Figure 2D), 2-eicosapenaenoyl-glycerol (2-EPG, Figure 2E), 2-docosapentaenoyl-glycerol (2-DPG, Figure 2F) and 2-docosahexaenoyl-glycerol (2-DHG, Figure 2G). The concentration of all of these 2-MAGs was comparable between raw DM, HoP-DM and HHP-DM (Figures 2A–G), demonstrating that human milk pasteurization using these two methods did not affect MAG levels.

## 4 Discussion

In this study, we found a better preservation of some eCBome mediators including four NAEs (AEA, PEA, OEA and LEA) in DM after HHP compared to HoP-DM. We observed that the concentrations of AEA, PEA, OEA and LEA was comparable in HHP-DM compared to raw DM, whereas after HoP their concentrations were significantly increased. We also found that DHEA (another NAE) and the seven MAGs analyzed herein were not affected by these two treatments of DM.

Arachidonic acid (ARA) is the most predominant long-chain polyunsaturated fatty acid in human milk (2, 3). ARA serves as a precursor to eCBs such as AEA, a neuromodulator activating the CB1/CB2 receptors in the central nervous system and peripheral tissues, and plays a key role in appetite and food intake (2–4). In addition to AEA, the presence of other co-occurring eCB-like metabolites (such as PEA, OEA, LEA, DHEA, 2-PG, 2-OG, 2-DHG and 2-EPG, which we also quantified in the present study) was shown to exhibit cannabimimetic responses through the enhancement of the activity of AEA (3–5). For instance, PEA and OEA were shown to reduce enzymatic breakdown, cellular uptake, and degradation of AEA (27). These metabolites may interfere with enzymatic activity as they can also act as substrates for catabolic and anabolic enzymes and thus in fine modulate eCBome mediator activation or deactivation (27). Perhaps more importantly, these eCBome mediators have their own molecular targets, such as, for example, peroxisome proliferator-activated receptor-α, transient receptor potential vanilloid type-1 channels and G-protein-coupled receptor-119, all of which are directly or indirectly involved in the control of food intake and body weight (28). Although, the activation of the CB1 receptor was shown to play a critical role in initiating milk suckling and in the control of body growth during early stages of life (8–10), at present, there is no full understanding of the roles of eCBs in human milk and further studies are needed to unravel their precise biological roles in infants.

In the present study, we did not observe that HoP and HHP processing modulate milk ARA levels (data not shown), which is the ultimate biosynthetic precursor of AEA and 2-AG. However, AEA originates from a complex metabolism of ARA-containing phospholipids (27–29). This biochemical pathway, with the corresponding fatty acid precursors instead of ARA, is also implicated in the biogenesis of PEA, OEA and LEA, which are also increased similarly to AEA in HoP-DM compared to raw DM and HHP-DM. Thus, we can postulate that HoP (treatment at 62.5°C for 30 min) may have stimulated some enzymes implicated in the biosynthesis of these NAEs. However, to our knowledge, no data are available concerning the presence of specific NAE biosynthesizing enzymes in human milk. Conversely, we can also postulate that these specific NAEs are more rapidly degraded in raw-DM and HHP-DM than in HoP-DM, suggestive of a possible inhibitory effect of HoP on catabolic NAE enzymes, which, again, have not been identified in human milk. These biochemical hypotheses will necessitate further *ad hoc* investigations. Yet, and in line with these hypotheses, HoP has been shown to alter numerous lipids in human milk, although conflicting results have been reported (21, 23, 30). Conversion of lipids, stimulation and/or inhibition of some enzymes have been proposed to explain the deleterious effects of HoP but no clear mechanisms have been demonstrated. Vincent et al. (31) has proposed that the deleterious effects of HoP could be attributed to the adherence of disrupted milk fat globules to container surfaces and to whether or not thermal treatment took place in the pasteurizer. Others studies have shown that temperature, storage conditions and duration drastically disturb the eCB metabolome in animal milk as well as in human plasma/serum, suggesting that eCBome mediators are highly sensitive to these factors (6, 32). For example, compared to human milk stored at −20°C, Wu et al. (32) demonstrated that milk stored at 4°C for one day exhibited dramatically increased concentrations of AEA, PEA, OEA and LEA, which are the NAEs also increased here in our HoP-DM samples. Thus, the thermal effect of HoP may probably be implicated in the differences between NAE concentrations observed in the present study. Finally, unsaturated eCBome mediators are also affected by non-enzymatic oxidative parameters (6, 32, 33). As the duration of HoP processing is 30 min, we cannot rule out an effect of oxidative stress on milk NAEs. In accordance with this hypothesis, we demonstrated in a recent study (34) that HoP treatment of DM significantly reduced the concentrations of γ-tocopherol, a powerful antioxidant, compared to raw-DM and HHP-DM, suggesting that the antioxidant capacity of HoP-DM is significantly reduced.

In sum, our data show that HoP-DM, which is routinely produced by HMBs for the feeding of preterm infants, contain higher levels of several eCBome mediators than HHP-DM. This finding raises now the following question: can these changes be harmful in preterm infants? In addition to the previously described CB1 receptor-mediated effects of eCBome mediators on infant suckling and appetite regulation, other physiological roles, due to the interaction with non-cannabinoid receptors, could also be affected such as, in pain-perception, neuronal development, energy metabolism or intestinal homeostasis, as milk eCBome mediators reach the gut first.

In preterm newborns, the intestine is a very vulnerable organ due to its weak intestinal barrier integrity (involved in mucus production, epithelial permeability as well as in gut immunity) (35, 36). Early intestinal alterations can lead to sepsis and necrotizing enterocolitis, one of the most prevalent and devastating inflammatory gastrointestinal disorders affecting 1%–12% of preterm infants (35, 36). The eCBome is known to play roles in immunity, inflammation, and in the interactions between microbiota and the host (37–39). For example, when the eCB system is activated, a disruption of the gut barrier, a low-grade inflammation and an increased adipogenesis are observed (37, 38). The increased gut permeability leads to the passage of lipopolysaccharides in the bloodstream, which further deranges gut barrier integrity, gut inflammation, thus finally affecting both the eCBome and other signalling systems not only in the intestine but also in the adipose tissue and other distal organs including the brain (37, 39–41). Collectively, our present data provide an additional way through which eCBome mediators, gut microbiota, adipose tissue development and intestinal function may interact in infants. In fact, we cannot rule out that the higher levels of some eCBome mediators in HoP-DM may have more marked deleterious effects on the immature intestine of preterm infants than HHP-DM but further clinical studies are needed to explore this hypothesis.

To conclude, compared to HoP, our HHP protocol allows a higher preservation of some major milk eCBome mediators that are bioactive lipids critical for the early postnatal development. Although future studies are needed to better understand the roles of these mediators in human milk, our study suggests that HHP-DM may be more suitable than HoP-DM to preserve the original composition of breast milk including the eCBome mediators.

## Data Availability

The original contributions presented in the study are included in the article/Supplementary Material, further inquiries can be directed to the corresponding author.

## References

[1] Grote V Verduci E Scaglioni S Vecchi F Contarini G Giovannini M European Childhood obesity project. Breast milk composition and infant nutrient intakes during the first 12 months of life. Eur J Clin Nutr. (2016) 70:250–6. 10.1038/ejcn.2015.162 26419197

[2] Hadley KB Ryan AS Forsyth S Gautier S Salem N. The essentiality of arachidonic acid in infant development. Nutrients. (2016) 8:216. 10.3390/nu8040216 27077882 PMC4848685

[3] Salem N Van Dael P. Arachidonic acid in human milk. Nutrients. (2020) 12:626. 10.3390/nu12030626 32121018 PMC7146261

[4] Witkamp RF. The role of fatty acids and their endocannabinoid-like derivatives in the molecular regulation of appetite. Mol Asp Med. (2018) 64:45–67. 10.1016/j.mam.2018.01.002 29325757

[5] Gaitán AV Wood JT Zhang F Makriyannis A Lammi-Keefe CJ. Endocannabinoid metabolome characterization of transitional and mature human milk. Nutrients. (2018) 10:1294. 10.3390/nu10091294 30213124 PMC6165354

[6] Gouveia-Figueira S Nording ML. Development and validation of a sensitive UPLC-ESI-MS/MS method for the simultaneous quantification of 15 endocannabinoids and related compounds in milk and other biofluids. Anal Chem. (2014) 86:1186–95. 10.1021/ac403352e 24377270

[7] Fradet A Castonguay-Paradis S Dugas C Perron J St-Arnaud G Marc I The human milk endocannabinoidome and neonatal growth in gestational diabetes. Front Endocrinol (Lausanne). (2024) 15:1415630. 10.3389/fendo.2024.1415630 38938519 PMC11208692

[8] Cascio MG. PUFA-derived endocannabinoids: an overview. Proc Nutr Soc. (2013) 72:451–9. 10.1017/S0029665113003418 24020830

[9] Fride E Ginzburg Y Breuer A Bisogno T Di Marzo V Mechoulam R. Critical role of the endogenous cannabinoid system in mouse pup suckling and growth. Eur J Pharmacol. (2001) 419:207–14. 10.1016/s0014-2999(01)00953-0 11426843

[10] Fride E Foox A Rosenberg E Faigenboim M Cohen V Barda L Milk intake and survival in newborn cannabinoid CB1 receptor knockout mice: evidence for a ‘CB3’ receptor. Eur J Pharmacol. (2003) 461:27–34. 10.1016/s0014-2999(03)01295-0 12568912

[11] Donvito G Nass SR Wilkerson JL Curry ZA Schurman LD Kinsey SG The endogenous cannabinoid system: a budding source of targets for treating inflammatory and neuropathic pain. Neuropsychopharmacol. (2018) 43:52–79. 10.1038/npp.2017.204 PMC5719110 28857069

[12] Fride E Gobshtis N Dahan H Weller A Giuffrida A Ben-Shabat S. The endocannabinoid system during development: emphasis on perinatal events and delayed effects. Vitam Horm. (2009) 81:139–58. 10.1016/S0083-6729(09)81006-6 19647111

[13] Warnier H Dauby J De Halleux V Denes S Emonts P Lefebvre C Prevention of prematurity’s complications. Rev Med Liege. (2024) 79:436–41. 38869136

[14] Haiden N Ziegler EE. Human milk banking. Ann Nutr Metab. (2016) 69(Suppl 2):8–15. 10.1159/000452821 28103607

[15] Picaud J-C Buffin R. Human milk-treatment and quality of banked human milk. Clin Perinatol. (2017) 44:95–119. 10.1016/j.clp.2016.11.003 28159212

[16] Pitino MA Unger S Doyen A Pouliot Y Aufreiter S Stone D High hydrostatic pressure processing better preserves the nutrient and bioactive compound composition of human donor milk. J Nutr. (2019) 149:497–504. 10.1093/jn/nxy302 30770541 PMC6398389

[17] Wesolowska A Sinkiewicz-Darol E Barbarska O Strom K Rutkowska M Karzel K New achievements in high-pressure processing to preserve human milk bioactivity. Front Pediatr. (2018) 6:1–10. 10.3389/fped.2018.00323 30519550 PMC6250976

[18] Demazeau G Plumecocq A Lehours P Martin P Couëdelo L Billeaud C. A new high hydrostatic pressure process to assure the microbial safety of human milk while preserving the biological activity of its main components. Front Public Health. (2018) 6:306. 10.3389/fpubh.2018.00306 30460221 PMC6232532

[19] Marousez L Tran L Micours E De Lamballerie M Gottrand F Pierrat V Metabolic hormones in human breast milk are preserved by high hydrostatic pressure processing but reduced by holder pasteurization. Food Chem. (2022) 377:131957. 10.1016/j.foodchem.2021.131957 34990954

[20] Tran LC Marousez L Micours E De Lamballerie M Thys L Gottrand F High hydrostatic pressure is similar to holder pasteurization in preserving donor milk antimicrobial activity. Pediatr Res. (2024) 95:1749–53. 10.1038/s41390-024-03022-9 38280953

[21] Tran LC Marousez L De Lamballerie M McCulloch S Hermann E Gottrand F The metabolome of human milk is altered differentially by holder pasteurization and high hydrostatic pressure processing. Front Nutr. (2023) 10:1107054. 10.3389/fnut.2023.1107054 36891163 PMC9987212

[22] Christian P Smith ER Lee SE Vargas AJ Bremer AA Raiten DJ. The need to study human milk as a biological system. Am J Clin Nutr. (2021) 113:1063–72. 10.1093/ajcn/nqab075 33831952 PMC8106761

[23] Ten-Doménech I Ramos-Garcia V Moreno-Torres M Parra-Llorca A Gormaz M Vento M The effect of holder pasteurization on the lipid and metabolite composition of human milk. Food Chem. (2022) 384:132581. 10.1016/j.foodchem.2022.132581 35257998

[24] Di Marzo V Sepe N De Petrocellis L Berger A Crozier G Fride E Trick or treat from food endocannabinoids? Nature. (1998) 396:636–7. 10.1038/25267 9872309

[25] Crozier Willi G Berger A Di Marzo V Bisogno T De Petrocellis L Fride E Lipids in neural function: modulation of behavior by oral administration of endocannabinoids found in foods. Nestle Nutr Workshop Ser Clin Perform Programme. (2001) 5:169–84. discussion 185-187. 10.1159/000061850 11510437

[26] Turcotte C Archambault A-S Dumais É Martin C Blanchet M-R Bissonnette E Endocannabinoid hydrolysis inhibition unmasks that unsaturated fatty acids induce a robust biosynthesis of 2-arachidonoyl-glycerol and its congeners in human myeloid leukocytes. FASEB J. (2020) 34:4253–65. 10.1096/fj.201902916R 32012340

[27] Fride E. The endocannabinoid-CB receptor system: importance for development and in pediatric disease. Neuro Endocrinol Lett. (2004) 25:24–30. 15159678

[28] Di Marzo V. New approaches and challenges to targeting the endocannabinoid system. Nat Rev Drug Discov. (2018) 17:623–39. 10.1038/nrd.2018.115 30116049

[29] Iannotti FA Di Marzo V Petrosino S. Endocannabinoids and endocannabinoid-related mediators: targets, metabolism and role in neurological disorders. Prog Lipid Res. (2016) 62:107–28. 10.1016/j.plipres.2016.02.002 26965148

[30] Gao C Miller J Middleton PF Huang Y-C McPhee AJ Gibson RA. Changes to breast milk fatty acid composition during storage, handling and processing: a systematic review. Prostaglandins Leukot Essent Fatty Acids. (2019) 146:1–10. 10.1016/j.plefa.2019.04.008 31186148

[31] Vincent M Ménard O Etienne J Ossemond J Durand A Buffin R Human milk pasteurisation reduces pre-lipolysis but not digestive lipolysis and moderately decreases intestinal lipid uptake in a combination of preterm infant *in vitro* models. Food Chem. (2020) 329:126927. 10.1016/j.foodchem.2020.126927 32516717

[32] Wu J Gouveia-Figueira S Domellöf M Zivkovic AM Nording ML. Oxylipins, endocannabinoids, and related compounds in human milk: levels and effects of storage conditions. Prostaglandins Other Lipid Mediat. (2016) 122:28–36. 10.1016/j.prostaglandins.2015.11.002 26656029

[33] Krishnamurthy HK Pereira M Rajavelu I Jayaraman V Krishna K Wang T Oxidative stress: fundamentals and advances in quantification techniques. Front Chem. (2024) 12:1470458. 10.3389/fchem.2024.1470458 39435263 PMC11491411

[34] Wemelle E Marousez L Lesage J De Lamballerie M Knauf C Carneiro L. *In vivo* assessment of antioxidant potential of human milk treated by holder pasteurization or high hydrostatic pressure processing: a preliminary study on intestinal and hepatic markers in adult mice. Antioxidants. (2022) 11:1091. 10.3390/antiox11061091 35739988 PMC9220199

[35] Hackam DJ Sodhi CP. Bench to bedside - new insights into the pathogenesis of necrotizing enterocolitis. Nat Rev Gastroenterol Hepatol. (2022) 19:468–79. 10.1038/s41575-022-00594-x 35347256

[36] Buckle A Taylor C. Cost and cost-effectiveness of donor human milk to prevent necrotizing enterocolitis: systematic review. Breastfeed Med. (2017) 12:528–36. 10.1089/bfm.2017.0057 28829161

[37] Muccioli GG Naslain D Bäckhed F Reigstad CS Lambert DM Delzenne NM The endocannabinoid system links gut microbiota to adipogenesis. Mol Syst Biol. (2010) 6:392. 10.1038/msb.2010.46 20664638 PMC2925525

[38] Cani PD Plovier H Van Hul M Geurts L Delzenne NM Druart C Endocannabinoids–at the crossroads between the gut microbiota and host metabolism. Nat Rev Endocrinol. (2016) 12:133–43. 10.1038/nrendo.2015.211 26678807

[39] Manca C Boubertakh B Leblanc N Deschênes T Lacroix S Martin C Germ-free mice exhibit profound gut microbiota-dependent alterations of intestinal endocannabinoidome signaling. J Lipid Res. (2020) 61:70–85. 10.1194/jlr.RA119000424 31690638 PMC6939599

[40] Cani PD Van Hul M. Gut microbiota in overweight and obesity: crosstalk with adipose tissue. Nat Rev Gastroenterol Hepatol. (2024) 21:164–83. 10.1038/s41575-023-00867-z 38066102

[41] Silvestri C Di Marzo V. The gut microbiome-endocannabinoidome axis: a new way of controlling metabolism inflammation, and behavior. Function (Oxf). (2023) 4:zqad003. 10.1093/function/zqad003 36778747 PMC9909364

